# Transcriptome Sequencing Reveals the Antiviral Innate Immunity by IFN-γ in Chinese Sturgeon Macrophages

**DOI:** 10.3389/fimmu.2022.854689

**Published:** 2022-03-17

**Authors:** Guangyi Ding, Chuwen Zheng, Bei Wang, Lifeng Zhang, Dan Deng, Qian Li, Huizhi Guo, Shuhuan Zhang, Qiaoqing Xu

**Affiliations:** ^1^ Institute of Chinese Sturgeon Disease, Yangtze University, Jingzhou, China; ^2^ Guangdong Provincial Key Laboratory of Pathogenic Biology and Epidemiology for Aquatic Economic Animals, Guangdong Ocean University, Zhanjiang, China; ^3^ Sturgeon Healthy Breeding and Medicinal Value Research Center, Guizhou University of Traditional Chinese Medicine, Guiyang, China

**Keywords:** *Acipenser sinensis*, IFN-γ, Illumina sequencing, immunoregulation, antiviral ability

## Abstract

To further study the biological function of interferon-gamma (IFN-γ) in the Chinese sturgeon (*Acipenser sinensis*), we conducted a transcriptome analysis of primary macrophages induced by IFN-γ using Illumina sequencing technology. We obtained 88,879 unigenes, with a total length of 93,919,393 bp, and an average length of 1,057bp. We identified 8,490 differentially expressed genes (DEGs) between the untreated and IFN-γ-treated macrophages, with 4,599 upregulated and 3,891 downregulated. Gene ontology (GO) analysis showed that 4,044 DEGs were enriched in the biological, cellular components, and molecular function categories. Kyoto Encyclopedia of Genes and Genomes (KEGG) identified 278 immunity-related pathways enriched for the DEGs. According to the GO enrichment results, eight key immunity-related genes were screened for verification using qPCR. Results indicate that IFN-γ can activate macrophage Interferon Regulatory Factors (IRFs) and type I interferon (IFN-I), activate RIG-I-like and Toll-like receptor-related pathways, and improve the antiviral ability of macrophages in Chinese sturgeon.

## Introduction

Interferon-gamma (IFN-γ) belongs to the type II interferon (IFN-II) family. IFN-γ is secreted by various cells, such as natural killer (NK cells), NK T cells, macrophages, bone marrow monocytes, T helper type 1 (Th1) cells, cytotoxic T lymphocytes (CTLs), and B cells (1). These cells are functionally divided into two groups that participate in innate or adaptive immune responses. IFN-γ production by NK cells and macrophages might play an important role in early defense against infection, while in adaptive immune responses, IFN-γ is mainly produced by lymphocytes and secreted by Th1 cells.

In contrast to mammals, in bony fish there are two types of IFN-γ. IFN-γ was found in Fugu (*Takifugu rubripes*) by (2), followed by IFN-γrel was described in zebrafish (3). The gene encoding Interferon-gamma related (IFN-γrel) might have been formed by tandem gene replication (4). Although IFN-γrel lacks a classical nuclear localization signal (NLS), it shares many characteristics with IFN-γ in terms of its protein structure, cell distribution, and immune response. Among all species with duplicate IFN-γ genes, the IFN-γrel gene has more structural similarities with known vertebrate IFN-γ genes. IFN-II gene is highly conserved in most fish and mammals, with a genetic structure of four exons and three introns, and several successive codons for arginine or lysine (5). IFN-γ and IFN-γrel have been identified in at least 20 species of fish, such as Fugu (*Takifugu rubripes*), zebrafish, grass carp (6) and *Anguilla japonica* (7). The sequence homology of IFN-γ in fish and mammals is generally low; however, the tertiary structure of IFN-γ appears to be conserved from fish to mammals and the gene is genetically homologous to higher vertebrates IFN-γ genes.

Belonging to the cytokine receptor family B (CRFB), IFN-γ receptors (IFN-γR) are very conserved in terms of gene structure and loci, as well as their protein functional domains or motifs (8). IFN-γR is composed of two transmembrane proteins, IFN-γR1 and IFN-γR2. IFN-γR1 is a ligand-binding chain, and IFNyR2 is a signal transduction chain. IFN-γR2 has a JAK-2 docking site P263PSIPLQIEEYL274 motif. IFN-γR1 possesses two functionally conserved residue sequences: L266PKS269 binding to JAK-1 and Y440DKPH444 interacting with STAT1 within the receptor’s intracellular domain (9). Unlike mammals, there are two copies of IFN-γR1 in bony fish, CRFB17(IFNGR1-1) and CRFB12(IFNGR1-2). The genes encoding the two IFN-γR1 receptors are located at different loci and exhibit conserved collinearity compared to their counterparts in other vertebrates. Two isoforms of IFN-γR1, called IFN-γR1-1and IFN-γR1-2, were described in goldfish and zebrafish, tissue expression analysis showed that IFN-γR1-1 expression was significantly higher than that of IFN-γR1-2 (10). In goldfish, IFN-γR1-1 is highly expressed in the kidneys and spleen, but IFN-γR1-2 is highly expressed in the brain. Microscopic binding studies showed that IFN-γ1 binds to IFN-γR1-1, but not to IFN-γR2, and IFN-γR1-2 preferentially binds to IFN-γrel (11). IFN-γR2, also known as CRFB6, has been identified in grass carp, rainbow trout, and Dabry’s sturgeon (12). In vertebrates, including humans, IFN-γR2 is encoded by a single-copy gene, linked to transmembrane protein 50B (TMEM50B) at the conserved site of class II cytokine receptors in vertebrates (9). IFN-γ signaling is caused by the binding of the active form of IFN-γ (a non-covalent homodimer) to a receptor containing complexes (IFN-γR1, IFN-γR2) that activate the intracellular Janus kinase/signal transducer and activator of transcription (JAK/STAT) signaling pathway and initiate the expression of multiple genes in the nucleus (10, 13).

IFN-γ is a typical Th1 cytokine that helps to activate macrophages and promote the Th1 response. Th1 immunity is an important immune defense mechanism against intracellular pathogens such as viruses and bacteria. IFN-γ also induces apoptosis, especially during viral infection, and inhibits cell proliferation (14–16). The antiviral function of IFN-γ has been explored in various fish, and can inhibit the proliferation of a variety of viruses in cells. For example, intramuscular injection of an IFN-γ expression plasmid in turbot (*Scophthalmus maximus*) inhibited the proliferation of viral hemorrhagic septicemia virus, VHSV, *in vivo* and effectively reduced mortality and the expression levels of a large number of pro-inflammatory factors and IFN-I is induced (17). In the Chinese black porgy (*Acanthopagrus schlegelii*), infection with red-spotted grouper nervous necrosis virus (RGNNV) not only induces IFN-γ expression in most tissues *in vivo*, but also significantly induces IFN-γ expression *in vitro*.

Meanwhile, Overexpression of IFN-γ in AsB cells (*Acanthopagrus schlegelii* brain cell) can effectively inhibit RGNNV proliferation in AsB cells and induce Mx1 and ISG15 expression (18, 19). In crucian carp, exposure of crucian carp Hematopoietic necrosis virus (CHNV) directly to infected gill tissue can induce IFN-γ significantly expression in gill and kidney and inhibit CHNV virus replication in gill and kidney (20). In the Chinese sturgeon, recombinant IFN-γ could effectively inhibit the pathological effect in endothelial progenitor cells (EPCs) infected with Spring viremia of carp virus (SVCV) and inhibit the expression of the P, G, and N genes of SVCV (21).

However, the mechanism of IFN-γ regulating the immune system of *Acipenser sinensis* remains unclear. This study used transcriptome sequencing to analyze differential gene expression in macrophages stimulated by IFN-γ. We found that IFN-γ significantly upregulated RIG-I and Toll-like receptor-related pathways and activated macrophages to form antiviral status.

## Materials and Methods

### Experimental Material

Chinese sturgeons (30–500g) were provided by the Yangtze River Fisheries Research Institute (Taihu Lake, Jingzhou, China). Recombinantly expressed Chinese sturgeon IFN-γ protein was preserved in our laboratory (21).

### Separation of Primary Macrophages

Chinese sturgeon was euthanized by MS-222, then the body surface of a Chinese sturgeon in a good growth state was swabbed with alcohol, and the head kidney and middle kidney were dissected. The excised kidneys were rinsed three times with 1 × phosphate-buffered saline (PBS). After cleaning, the kidneys were placed in 75% medical alcohol for 45 s. The tissues were ground and filtered into a 15 mL centrifuge tube. M199 complete medium (Invitrogen, Carlsbad, CA, USA) was added while grinding. After grinding, tissues were centrifuged at 500 × *g* for 15 min, and the supernatant was discarded. The centrifugation step was repeated twice, and the cells were resuspended in M199 complete medium. Three new 15 mL centrifuge tubes were taken, and 4 mL of 52% Percoll (Solarbio, Beijing, China) and 4 mL of 34% Percoll were added successively. Finally, the cell suspension was slowly added to the upper layer of 34% Percoll. The cells were centrifuged at 500 × *g* at room temperature for 50 min (with slow increases and decreases in the centrifuge speed). After centrifugation, the cells formed six layers, with the macrophages forming layer four. The supernatant was removed, and the fourth layer was slowly extracted into a new 15 mL centrifugation tube. M199 medium (10 mL) was added, the cells were centrifuged at 500 × *g* for 15 min, the supernatant was discarded, and M199 (containing 10% fetal bovine serum (FBS; Invitrogen)) was added for re-suspension. The cells were successively divided into six-well plates and cultured normally. When the cell coverage reached 80% and growth appeared normal, the experimental groups were treated with 100 ng/mL IFN-γ in PBS (labeled as AM 1, 2, and 3, respectively), and the control groups were treated with an equal volume of PBS (labeled as AMC 1, 2, and 3, respectively); the cells were collected after 24 h of induction.

### Total RNA Extraction and Quality Detection

The cells with a coverage rate of more than 80% of the 25 cm^2^ cell culture flask and in a good growth state were washed three times with 1 × PBS, the residual liquid was discarded, 1 ml of Trizol (Invitrogen) was added for lysis, and the cells were mixed by aspiration into a 1.5 ml RNase-free EP tube (Axygen, SFO, USA), and left at room temperature for 5 min to fully lyse. Then, 200 μl of chloroform (Sinopharm Chemical Reagent Co. LTD, Shenzhen, China) was added, the mixture was shaken vigorously for 30 s to mix the aqueous phase and organic phases left to stand at room temperature for 5 min, after centrifugation for 10 min at 10000 × *g* at 4°CC, the samples were divided into three layers (upper layer of RNA, middle layer of protein, and lower layer of cell debris). The upper aqueous phase was carefully removed into a new 1.5 ml RNase-Free EP tube, and pre-cooled isopropyl alcohol (Sinopharm Chemical Reagent Co. LTD) was added to the tube. After gentle and full mixing, the tube was stored at -20°CC for 15 min. The supernatant was discarded after centrifugation at 10000 × *g* at 4°CC for 10 min. Pre-cooled 75% ethanol (Sinopharm Chemical Reagent Co. LTD) was added to wash the precipitate 1-2 times (with centrifugation for 10 min at 7500 × *g* and 4 °CC; after adding 75% ethanol, it was only necessary to gently reverse the EP tube to precipitate and float the RNA). Put the RNA precipitation into the ultra-clean workbench, turn on the fan and blow for 2-3 min, so that the alcohol can be quickly swept away. DiethyI pyrocarbonate (DEPC; Takara, Dalian, China) water was added to dissolve the precipitate. The RNA concentration and quality in the samples were determined using a spectrophotometer and agarose gel electrophoresis, and the RNA was stored at −80 °CC for later use. High-quality RNAs were used to synthesize cDNA for quantitative real-time PCR.

### Construction of cDNA Libraries

After DNA digestion using DNase, the mRNA was enriched using Oligo(dT) magnetic beads. The fragmentation buffer was added to break the mRNA into short fragments. Reverse transcription PCR and random six-base primers were used to generate first-strand cDNA using the fragmented mRNA as a template, followed by a second-strand synthesis reaction to produce double-stranded cDNA, which was purified using PCR cleanup and gel extraction kits (Takara). After end repair and the addition of poly(A), the short fragments were linked with sequencing adapters, PCR amplification was performed after the fragment size was selected. After the constructed libraries were quantified with Agilent 2100 Bioanalyzer (Agilent, Santa Clara, CA, USA), they were sequenced using Illumina HiSeq 2500 sequencer (Illumina, San Diego, CA, USA) to produce 125 bp or 150 bp paired-end reads.

### Data Preprocessing, Quality Control, and Assembly

Raw reads in the FASTA format sequences were subjected to quality filtration to obtain high-quality reads for subsequent analysis. Firstly, Trimmomatic software (22) was used for quality control and removal of adapters. Low-quality bases and N bases were filtered out to obtain high-quality clean reads. Trinity (version: 2.4) (23) was used to splice the clean reads to produce transcript sequences using the paired-end method. The longest transcript was selected as a unigene according to their sequence similarity and length. Then, CD-HIT software (24) clustering was used to remove redundancies to obtain a final set of unigenes, which was used as the reference set for subsequent analysis.

### Unigene Functional Annotation

Using BLASTX (http://www.ncbi.nlm.nih.gov/BLAST/) with an E-value threshold of 1e-5 performed the unigene functional annotation. The databases being utilized include non-redundant (NR) (http://www.ncbi.nlm.nih.gov), Clusters of Orthologous Groups (COG)/eukaryotic Orthologous Groups (KOG) (http://ftp://ftp.ncbi.nih.gov/pub/COG/KOG/), and Swiss-Prot protein (http://www.expasy.ch/sprot) databases. Based on the result of Swiss-Prot, we mapped Swiss-Prot IDs to gene ontology (GO) terms to obtain GO annotation of the unigenes. Finally, the unigenes were subjected to Kyoto Encyclopedia of Genes and Genomes (KEGG) (25) using diamond software (26), and HMMER (27) was used to compare the Pfam database (28) for functional analysis of unigene.

### Unigene Quantification, Differential Unigene Screening, Functional Enrichment, and Cluster Analysis

The FPKM (fragments per kilobase of transcript per million mapped reads) and count values of the unigene were analyzed using bowtie2 (29) and eXpress (30). The DEGs were identified using the DESeq functions estimate Size Factors and nbinom Test (31). DEGs (*P*-value < 0.05 and foldchange > 2) were picked out, and DEGs GO enrichment and KEGG pathway enrichment analyses were performed. Unsupervised hierarchical clustering of the DEGs was carried out, and their expression patterns among different samples were displayed in the form of heat maps.

### Identification and Expression Analysis of Immunity-Related Genes

The transcript levels of eight immunity-related genes (*As*IFN-e1-3, *As*IFN-γ, IRF1, 2, 3, and 7) were screened using quantitative real-time RT-PCR (qPCR) for verification. The specific primers are shown in **Supplementary Table 1**.

## Results

### Transcriptome Data Statistics and *De Novo* Assembly

**Figure 1:**
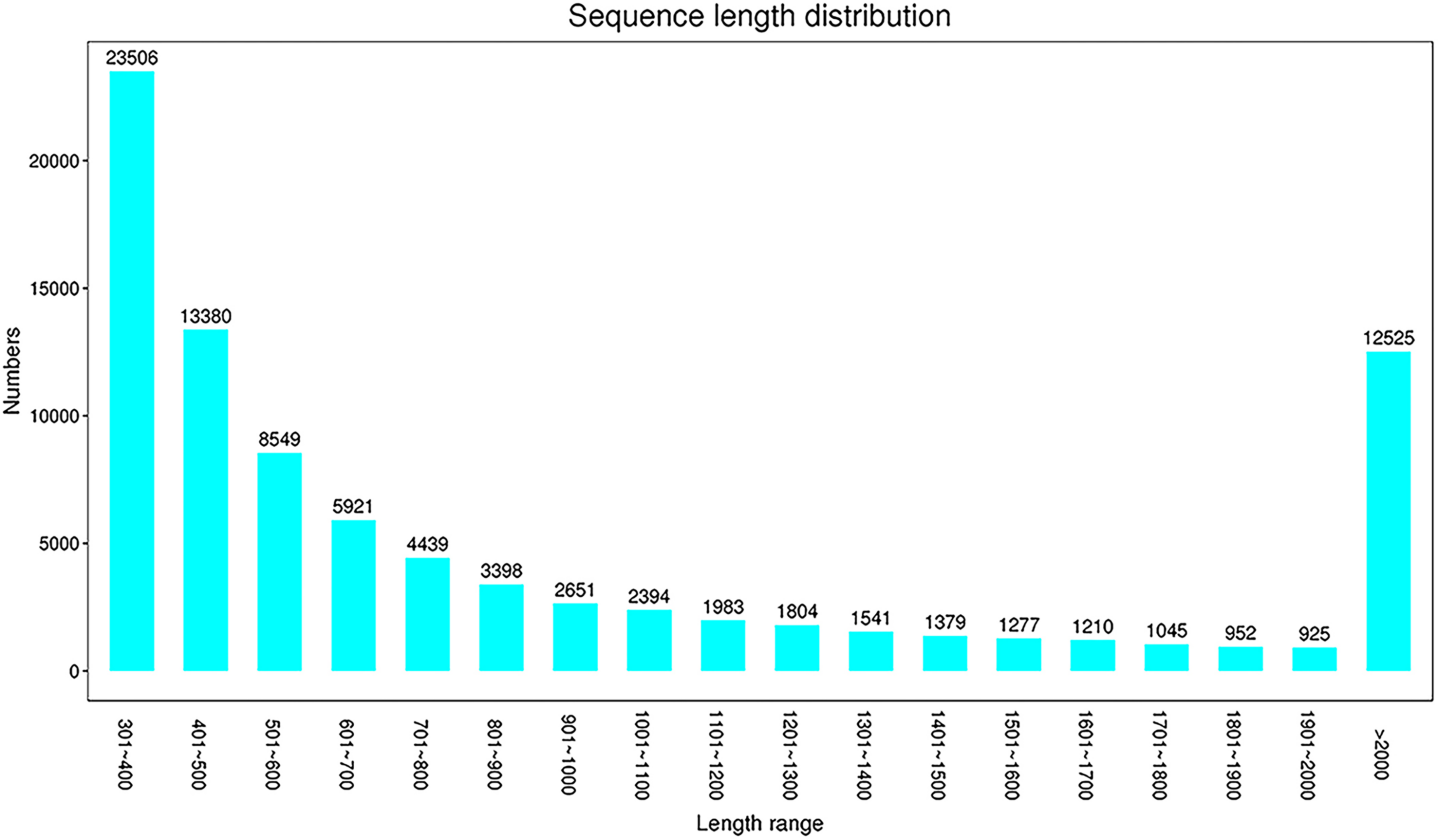
Unigene Length distribution. X-axis is the sequence length range, Y-axis is the number.

Raw reads were obtained by high-throughput sequencing, and 268,120,424 and 280,376,796 reads were detected in the experimental group (AM 1, 2, and 3) and the control group (AMC 1, 2, and 3), respectively. After further quality control and filtering of raw reads, high-quality clean reads were obtained by Trimmomatic. There were 272,115,614 reads in the AM group and 273,895,990 reads in the AMC group. The average sequences numbers in the AM and AMC group were 90,705,204 and 210,075,147, respectively. The Q30 of the original data for each sample was 92.99-93.28%, the effective data volume was 12.00-13.40 G, and the average GC content was 46.29% (**Supplementary Table 2**). The filtered clean reads were spliced into 88,879 unigenes, with a total length of 93,916,393 bp, a longest length of 26,838 bp, an average length of 1,057 bp, and an N 50 value of 1,727 bp (**Supplementary Table 3**). The main length distribution was 23,506 (301~400 bp), 13,380 (401~ 500 bp) and 12,525 (>2,000 bp) (**Figure 1**). The above data indicated that the library of primary macrophages induced by Chinese sturgeon IFN-γ is reliable and can be further studied and analyzed.

### Unigene Function Annotation

**Figure 2:**
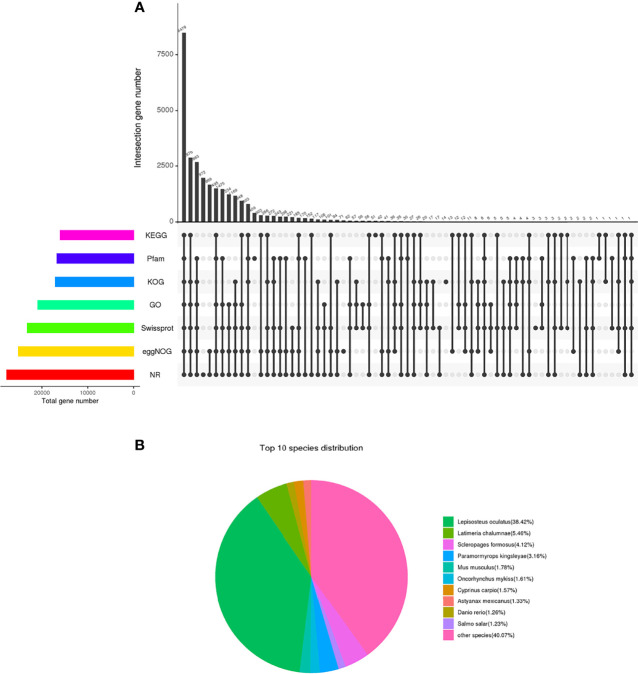
The database annotation results of the unigene. **(A)** Venn diagram of each database annotation. the number on the top of the bar represents the result of the intersection of the databases with black dots in the following matrix, and the column on the left represents the number of unigene annotated in all databases. **(B)** Species comparison distribution of unigenes in NR database.

The database annotation results of the unigene were as follows: 27679 (31.14%) genes were annotated to the NR database; 23190 (26.09%) genes were annotated to the Swiss-Prot database; 16011 (18.01%) genes were annotated to the KEGG database; 17081 (19.22%) genes were annotated to the KOG database; 25138 (28.28%) genes were annotated to the eggNOG database; 20900 (23.52%) genes were annotated to the GO database; and 16700 (18.79%) genes were annotated to the Pfam database (**Figure 2A** and **Table 4**). The annotation distribution of NR species showed that the similarity between Chinese sturgeon and *Lepisosteus oculatu* in terms of genomic homology and phylogenetic analysis was the highest, which was consistent with the homology rate of 38.42% of *Lepisosteus oculatus* in this experiment. *Latimeria chalumnae*, *Scleropages formosus*, and Chinese sturgeon all belong to the Osteichthyes, which occupy an extremely important position in the evolutionary history from fish to vertebrates, the homology rates between the two species and the Chinese sturgeon were 4.12% and 5.46%, respectively. Chinese sturgeon, *Cyprinus carpio*, *Salmo salar*, and zebrafish belong to the Actinopterygii, among which, *Cyprinus carpio*, *Salmo salar* and Chinese sturgeon are similar to each other and have a migratory life habit. As a model organism, zebrafish has a higher genetic homology with human, up to 87% (**Figure 2B**). Therefore, the homology of Chinese sturgeon with *Cyprinus carpio*, *Salmo salar* and *Oncorhynchus mykiss* was 1.57%, 1.23%, and 1.61%, respectively. In addition, 40.07% of unigene were homologous to other vertebrate genes.

### Analysis of Differentially Expressed Genes

**Figure 3:**
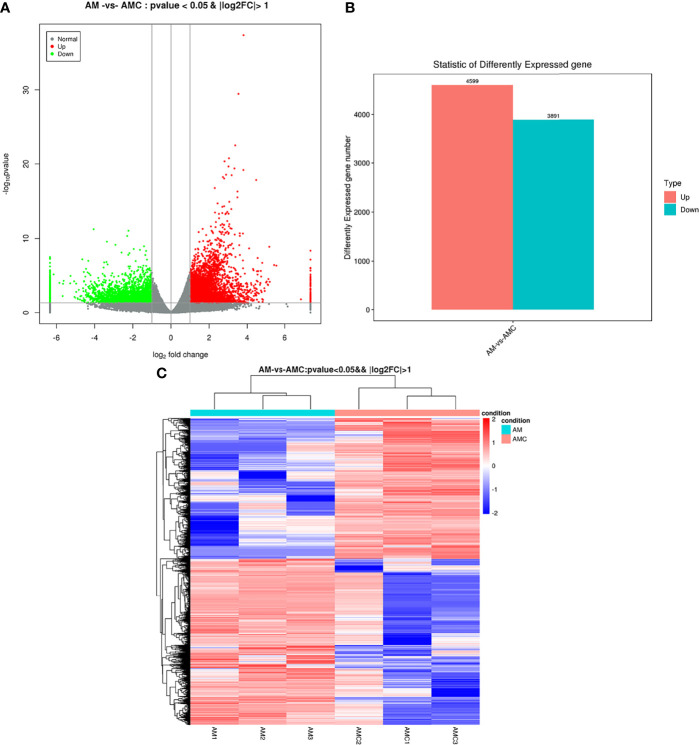
Analysis results of differentially expressed genes. **(A)** Distribution of DEGs volcanoes. X-axis is the display of log2 foldchange, Y-axis is -log_10_ *P*-value; Red represents upregulation unigene, green represents downregulation unigene, gray represents non differentiation unigene. **(B)** Statistical chart of the number of DEGs. The abscissa is DEGs, and the ordinate is the number of difference genes; Red represents upregulation unigene, green represents downregulation unigene. **(C)** Clustering diagram of different groups. Red indicates high expression of unigene and blue indicates low expression of unigene.

A total of 8490 DEGs were screened out. The distribution of DEGs was represented by a volcano map (**Figure 3A**). Among the DEGs between the experimental group (AM) compared with the control group (AMC) 4599 were upregulated, 3891 were downregulated, and with false discovery rate value of *P* < 0.05 & | log_2_FC | > 1 (**Figure 3B**). There may be differences between different groups under the same experimental conditions. Therefore, we analyzed the correlations among the DEGs in the three replicates in each group, which showed a strong correlation among the AM1, 2, and 3, and most of the genes with high expression were clustered in the same branch. AMC1 and AMC3, but not AMC2, showed very strong correlation in the control group. In addition, the correlation between genes in the AM group and those in the AMC group was low and the difference was large (**Figure 3C**), indicating that the screened DEGs were reliable and convincing.

### GO Enrichment Analysis of DEGs

**Figure 4:**
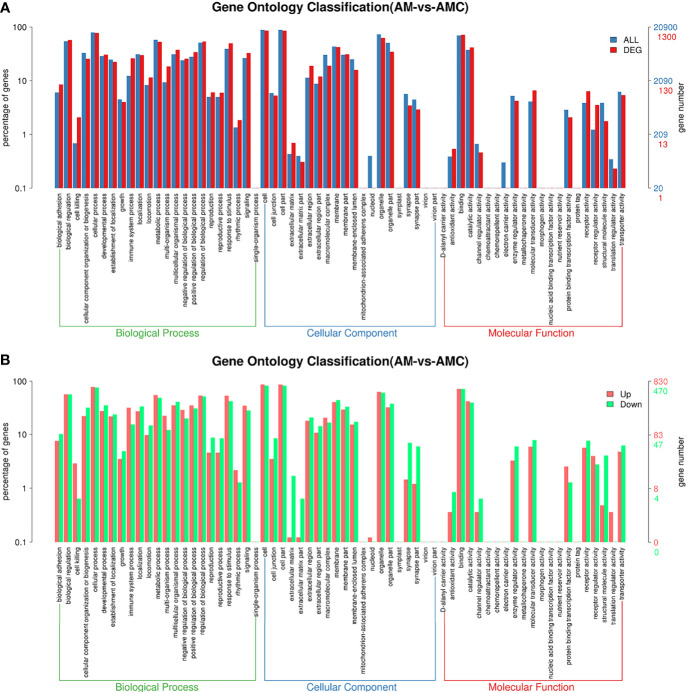
Differential unigenes were analyzed by GO enrichment. **(A)** Comparison of distribution of differentially expressed unigene and all unigene at GO level2. Blue represents all unigene enriched GO level 2 items, red represents differential unigene enriched GO level 2 items, the horizontal axis represents the items name, and the vertical axis represents the number and percentage of unigene of corresponding items. **(B)** Comparison of upregulation and downregulation of unigene at GO level 2. Red indicates up regulation of go level 2 items enriched by DEGs, blue indicates down regulation of go level 2 items enriched by DEGs, the horizontal axis is the item name, and the vertical axis is the number and percentage of unigene of corresponding items.

After the DEGs were selected, GO enrichment analysis was performed to determine which biological processes were mainly affected by the DEGs. 4004 DEGs were detected in the GO enrichment, 1300 GO annotations were obtained, 830 were upregulated, and 470 were downregulated. The unigene annotated by the GO database can be divided into biological processes (biological processes, BP), Cellular Component (CC), molecular function (MF). The GO level 2 results were further divided into 64 subcategories. BP was divided into 23 subcategories, among which the three categories with the largest number of DEGs were: cellular process (77.54%), regulation of biological process (53.61%), metabolic process (53.08%); CC was divided into 20 subgroups, and the components with the largest proportion of DEGs were: cell (85.69%), cell part (85.38%), organelle (62.69%), membrane (42.23%); MF was divided into 21 subtypes, and the three most important ones were: binding (71.92%), catalytic activity (41.69%), molecular sensing activity (41.69%) (**Figure 4A**). The differences between the enriched DEGs and all unigenes in BP, CC, and MF were compared and analyzed to maximize the visualization of the regulatory effect of Chinese sturgeon IFN-γ on the transcription of Chinese sturgeon in GO enrichment function annotation. In BP, the expression rate of DEGs enriched in biological functions such as cell process, metabolic process, biological regulatory process, and stimulus-response was greater than 50%; in CC, the differential expression rate of DEGs in cells, cell parts and organelles was the highest; in MF, the expression rate was the highest only in the binding process (**Figure 4B**). The results indicated that Chinese sturgeon IFN-γ is involved in the three biological processes of BP, CC, and MF, and had a certain immune regulation effect in all three categories.

### KEGG Enrichment Analysis of the DEGs

**Figure 5:**
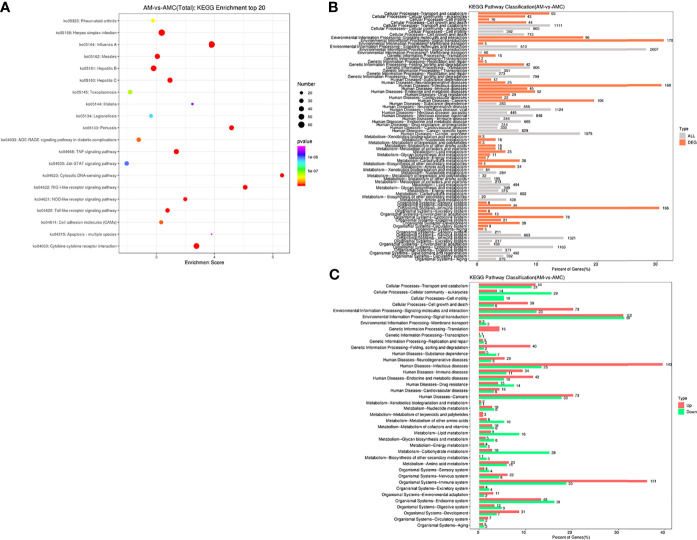
Enrichment analysis of differential gene Unigene. **(A)** KEGG enrichment top20 pathway bubble chart. The X-axis is the enrichment score, the bubble size indicates the number of unigene contained in the item, and the color of the bubble from purple to red indicates *P*-value. **(B)** Distribution comparison of DEGs and all unigene at KEGG level 2. The vertical axis represents the name of level 2 pathway, and the number to the right of the column represents the number of DEGs annotated to that level 2 pathway. **(C)** Distribution of upregulated and downregulated unigene at KEGG level 2.

KEGG is a major public database related to pathways. Pathway entries with significantly enriched unigenes were found through Pathway analysis of DEGs, providing clues as to which cell pathways might be related to the differences in unigene expression between the samples. In this study, 16011 unigenes annotated by KEGG were combined, 1300 DEGs were enriched for 278 pathways, 222 pathways were significantly downregulated, and 248 pathways were significantly upregulated. The top 20 pathways with the largest number of DEGs were screened. The -log_10_ *P*-value corresponding to each entry was sorted from large to small (**Figure 5A** and **Supplementary Table 5**). Among them, six signaling pathways related to interferon regulation were identified: The RIGI-like receptor signaling pathway (35 unigenes) and Cytosolic DNA-Sensing Pathway (28 unigenes), NOD-like receptor signaling pathway (41 unigenes), Toll-like receptor signaling pathway (TLR) (27 unigenes), and the JAK-STAT signaling pathway (16 unigenes). Pathways enriched for DEGs and all unigenes at KEGG Level 2 level were divided into six categories: Organic systems (5297, 57.05%), metabolism (3027, 32.59%), human diseases (7187, 77.4%), genetic information processing (2229, 24.01%), environmental information processing (3277, 35.3%), cellular processes (3021, 32.54%) (**Figure 5B**). In terms of the expression trends of the unigenes, 143 unigenes were upregulated in human infectious diseases, followed by 131 unigene that were upregulated in the immune system of the organic system, and 58 unigenes were downregulated in signal transduction in environmental information processing (**Figure 5C**). These results indicated that the recombinant IFN-γ protein of Chinese sturgeon could regulate the immune pathways of Chinese sturgeon to exert its related biological functions, and these data provide a certain theoretical basis for further in-depth studies of the mechanism of IFN-γ related to immune regulation of the Chinese sturgeon.

### Identification and Expression Analysis of Immunity-Related Genes

**Figure 6:**
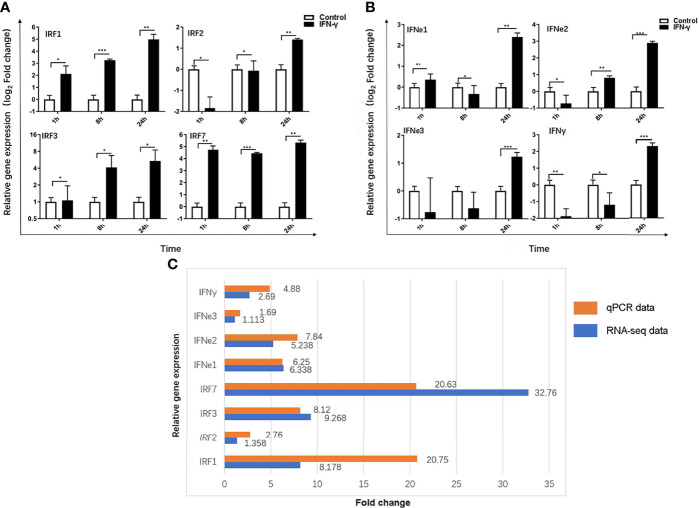
Immunity-related genes expression induced by IFN-γ of Acipenser sinensis. **(A)** Relative expression of IRF1, 2, 3 and 7 induced by IFN-γ of Acipenser sinensis. **(B)** Relative expression of IFN–γ, IFN-e1,2,3 induced by IFN - γ of Acipenser sinensis. **(C)** Fold change of 8 immune genes DEG screened by qPCR and transcriptome. The vertical axis represents the name of the gene and the horizontal axis represents the multiples, red represents qPCR data and blue represents transcriptome data results. *P*-values of less than 0.05 were considered statistically significant (*P<0.05, **P<0.01, ***P<0.001).

To further analyze the function of IFN-γ in Chinese sturgeon and verify the transcriptome data, eight immunity-related genes were screened from the transcriptome, namely, *As*IFN-e1, 2, 3, *As*IFN-γ, IRF1, 2, 3, and 7, and analyzed using qPCR. The results showed that IFN-γ could induce the expression of IRF1, 2, 3, and 7 at 1, 8, and 24 h with the same significantly upregulated trend as observed in the transcriptome data. From 1 h to 24 h, their expression increased to a maximum of 20.75 times, 2.76 times, 8.12 times, and 20.63 times, respectively (**Figure 6A**). *As*IFN- e1, 2, 3, and *As*IFN-γ were induced in Chinese sturgeons. Except for *As*IFN-e1, the expression of *As*IFN-e2, 3, and *As*IFN-γ showed the same upregulation trend as observed in the transcriptome data. The expression levels of *As*IFN-e1, 2, 3, and *As*IFN-γ were all lower at 1 h; however, IFN-e1, 2, 3, and IFN-γ expression levels were all upregulated to the highest degree at 24 h, by 6.25 times, 7.84 times, 1.69 times, and 4.88 times respectively (**Figure 6B**); In addition, qPCR results were similar to the up-regulation multiple of transcriptome differential genes (**Figure 6C**). The above results indicated that *As*IFN-γ induced primary macrophages of Chinese sturgeons and had strong immunomodulatory effects on interferon (*As*IFN-e1,2,3 and *As*IFN-γ) and interferon regulatory factors (IRF1, 2, 3, and 7). The qPCR results were similar to the transcriptome data, within the range of allowable error, demonstrating the transcriptome data reliability and validity.

## Discussion

Accurately describing the signaling pathways that cause gene expression and activation, and fully exploring the structure, classification, and function of the transcriptome information. As an effective technique to study gene expression and analyze differentially expressed genes and their functions, high-throughput sequencing has been used widely to study a variety of vertebrates, such as soiny mullet (*Liza haematocheila*), grass carp (*Ctenopharyngodon idella*), orange-spotted grouper (*Epinephelus coioides*), and Siamese fighting fish (*Betta Splendens*) (32–35). Based on the protection of genetic resources of the endangered species Chinese sturgeon and the deep exploration of the biological function of IFN-γ in Chinese sturgeon, this study described the effect of IFN-γ on primary macrophages of Chinese sturgeon at 24 h, and analyzed the expression changes of IFN-γ-related, immunity-associated genes and pathways, which is crucial to understanding the immune mechanism of IFN-γ.

Through transcriptomic analysis, 888879 unigene were obtained, 8490 DEGs were screened, and 4004 DEGs were enriched in the GO analysis, many of which were involved in immune-related processes, such as immune response, innate immune response, regulation of apoptosis process, inflammatory response, cytokine-mediated signaling pathway, and viral defense response. These results indicate that IFN-γ has strong immune stimulation or immunomodulatory ability on macrophages. Meanwhile, KEGG annotation results showed that almost all IFN-γ induced DEGs were significantly associated with the three most common pathways, including the immune system, cellular processes, and metabolism. Further analysis of 278 KEGG enriched signaling pathways identified the main pathways as the TNF signaling pathway, the chemokine signaling pathway, the RIG I-like receptor signaling pathway, and the TLR signaling pathway. qPCR verified that the expression patterns of immune pathway-related DEGs were consistent with the transcriptomic results, indicating that the transcriptomic sequencing results were reliable.

TLRs and RIG-I are key molecules to identify pathogens such as ssRNA/dsRNA virus, LPS, Cytosolic DNA, which can be detected in most tissues (36). Recombinant IFN-γ induced upregulation of toll-like receptors TLR2, TLR7, TLR8, and TLR13 in macrophages. In mice, TLR2 plays an important role in Listeria resistance, including enhancing the phagocytosis of macrophages, promoting the production of TNF-α, IL-12, and NO, and promoting the expression of costimulatory molecules CD40 and CD60 (37). TLR7 and TLR8 play a critical role in sensing viral ssRNA in the endosome, in which TLR13 specifically recognizes single-stranded RNA. After recognizing their respective PAMPs, TLRs recruit a set of specific adaptor molecules containing TIR domains, such as MyD88, and initiate downstream signaling events, leading to the secretion of inflammatory cytokines, type I IFN, chemokines, and antimicrobial peptides, the recruitment of neutrophils, the activation of macrophages, and the induction of interferon-stimulating genes, thus killing the infected pathogen directly (38). In this experimental group, TLR4, TRAF6, P38, and AP1 were down-regulated in different degrees, indicating that IFN-γ also had a certain degree of inhibition on TLR4-activated related genes in Chinese sturgeons. In human macrophages, IFN-γ selectively suppresses a subset of TLR4-activated genes and enhancers to potentiate macrophage activation. RIG-I receptor-related genes RIG-I, MDA5, and TRIM25 were also significantly upregulated. As the primary cytoplasmic RNA monitoring mechanism, the RIG-I-MAVS signaling pathway has been intensively studied as a natural immune protection system for antiviral infection (39). RIG I-like receptors (RLRs), including RIG-I, MDA5, and LGP2, are a series of cytoplasmic RNA helicases that detect the accumulation of multiple viral RNAs during viral infection or replication (40). In most cell types except pDCs, RLRs are essential for antiviral responses. Thus, IFNγ maintains antiviral status in macrophages through RIG-I and Toll-like receptor-related signaling pathways and up-regulation of related enzymes.

Interestingly, Chinese sturgeon IFN-γ can also induce the expression of a large number of C-X-C motif family genes, and these chemokines are involved in the activation, adhesion, and recruitment of leukocytes to inflammatory sites, promoting immune response, stem cell survival, and angiogenesis by binding to G protein-coupled cell surface receptors. Normally undetectable in most non-lymphoid cells, which IFN-γ can strongly induce during infection or inflammation. In mice, IFN-γ can induce the expression of CXCL10 significantly in primary macrophages, reaching a peak at 3–6 h; IFN-γ in rainbow trout can also stimulate the significant expression of CXCL9, CXCL10, and CXCL11 in RTS-11 cells. In addition, the combination of IFN-γ and lipopolysaccharide, IL1β or TNFα produces a synergistic effect in a variety of cell types, which strongly induce the expression of various chemokines (41). In an *in vitro* study of rainbow trout IFN-γ, TNFα combined with IFN-γ induced the expression levels of CXCL9, CXCL10, and CXCL11 in RTS11 cells to more than 50 times higher than that induced by TNFα alone (42).

The result of stimulation of *Acipenser dabryanus* with LPS and polyI:C, type I interferon *Ad*IFNe1, e2 and e3 showed higher antiviral activity than *Ad*IFN-γ, *Ad*IFN-γ showed more antibacterial than type I interferon. The amino acid sequence similarity between (*Ad*IFNe1 and *As*IFNe1, *Ad*IFNe2 and *As*IFNe2, *Ad*IFNe3 and *As*IFNe3, *Ad*IFN-γ and *As*IFN-γ) are more than 95%, which indicated that the two types interferon functions of these two species were very similar.

In this study, transcriptomic data on many key genes and pathways were obtained, revealing that IFN-γ induces the expression of relevant immune genes or proteins, activates intracellular signaling pathways, regulates cellular and humoral immune systems, and is involved in the regulation of metabolism. These data provide a theoretical reference for further study of the biological function of IFN-γ in Chinese sturgeon and the analysis of the biological evolution and immune gene evolution of ancient Chinese sturgeon species.

## Data Availability Statement

The datasets presented in this study can be found in online repositories. The names of the repository/repositories and accession number(s) can be found below: https://www.ncbi.nlm.nih.gov/sra; PRJNA801715.

## Ethics Statement

The animal study was reviewed and approved by Animal Ethics Committee of Yangtze University.

## Author Contributions

GD contributed to the writing of the manuscript. GD and CZ contributed to the data analysis. LZ, DD, QL, and HG contributed to laboratory work. BW and SZ contributed to the text editing. QX contributed to the conception of the idea and design. All authors read and approved the manuscript.

## Funding

This work research was financially supported by the Research Fund Program of Guangdong Provincial Key Lab of Pathogenic Biology and Epidemiology for Aquatic Economic Animals (No. PBEA2021ZD03) and the Basic Research Project of Guizhou Province.

## Conflict of Interest

The authors declare that the research was conducted in the absence of any commercial or financial relationships that could be construed as a potential conflict of interest.

## Publisher’s Note

All claims expressed in this article are solely those of the authors and do not necessarily represent those of their affiliated organizations, or those of the publisher, the editors and the reviewers. Any product that may be evaluated in this article, or claim that may be made by its manufacturer, is not guaranteed or endorsed by the publisher.

## Supplementary Material

Supplementary Table 1 Nucleotide sequences of primers.

Supplementary Table 2 Transcriptome data statistics.

Supplementary Table 3 Clean reads assembly statistics.

Supplementary Table 4 Statistical table of database annotation.

Supplementary Table 5 Pathway enrichment analysis of AM-vs-AMC, statistical table of KEGG enrichment’s top 20 signal pathways.

Supplementary Table 6 Immune signal pathway and screening of differential genes.

## References

[1] Li L Chen SN Laghari ZA Huang B Huo HJ Li N. Receptor Complex and Signalling Pathway of the Two typeII IFNs, IFN-γ and IFN-γrel in Mandarin Fish or the So-Called Chinese Perch Siniperca Chuatsi. Dev Comp Immunol (2019) 97:98–112. doi: 10.1016/j.dci.2019.03.016 30922782

[2] Zou J Yoshiura Y Dijkstra JM Sakai M Ototake M Secombes C. Identification of an Interferon Gamma Homologue in Fugu, Takifugu Rubripes. Fish Shellfish Immunol (2004) 17(4):403–9. doi: 10.1016/j.fsi.2004.04.015 15312667

[3] Igawa D Sakai M Savan R. An Unexpected Discovery of Two Interferon Gamma-Like Genes Along With Interleukin (IL)-22 and -26 From Teleost: IL-22 and -26 Genes Have Been Described for the First Time Outside Mammals. Mol Immunol (2006) 43(7):999–1009. doi: 10.1016/j.molimm.2005.05.009 16005068

[4] Savan R Ravichandran S Collins JR Sakai M Young HA. Structural Conservation of Interferon Gamma Among Vertebrates. Cytokine Growth Factor Rev (2009) 20:115–24. doi: 10.1016/j.cytogfr.2009.02.006 PMC2755191 19268624

[5] Zou J Tafalla C Truckle J Secombes CJ. Identification of a Second Group of Type I IFNs in Fish Sheds Light on IFN Evolution in Vertebrates. J Immunol (2007) 179(6):3859–71. doi: 10.4049/jimmunol.179.6.3859 17785823

[6] Chen WQ Xu QQ Chang MX Zou J Secombes CJ Peng KM. Molecular Characterization and Expression Analysis of the IFN-Gamma Related Gene (IFN-γrel) in Grass Carp *Ctenopharyngodon Idella* . Vet Immunol Immunopathol (2010) 134(3-4):199–207. doi: 10.1016/j.vetimm.2009.09.007 19800136

[7] Li X Huang WS Huang B Xu JS Zhai SW Liang Y. Prokaryotic Expression and Purification of Type II Interferon Genes (IFN-γ and IFN-γ REL) From Japanese Eel. Mar Fish (2019) 5:567–77. doi: 10.13233/j.cnki.mar.fish.2019.05.004

[8] Qi JW Tang N Wu YB Chen H Wang SY Wang B. The Transcripts of CRF and CRF Receptors Under Fasting Stress in Dabry’s Sturgeon (*Acipenser Dabryanus* Dumeril). Gen Comp Endocrinol (2019) 280:200–8. doi: 10.1016/j.ygcen.2019.05.005 31075270

[9] Chen SN Huang B Zhang XW Li Y Zhao LJ Li N. IFN-γ and Its Receptors in a Reptile Reveal the Evolutionary Conservation of Type II IFNs in Vertebrates. Dev Comp Immunol (2013) 41(4):587–96. doi: 10.1016/j.dci.2013.07.002 23850722

[10] Bach EA Aguet M Schreiber RD. The IFN Gamma Receptor: A Paradigm for Cytokine Receptor Signaling. Annu Rev Immunol (1997) 15:563–91. doi: 10.1146/annurev.immunol.15.1.563 9143700

[11] Mikulecký P Cerný J Biedermannová L Petroková H Kuchař M Vondrášek J. Increasing Affinity of Interferon-Receptor 1 to Interferon-By Computer-Aided Design. BioMed Res Int (2013) 2013:752514. doi: 10.1155/2013/752514 PMC3807708 24199198

[12] Johnson HM Noon-Song EN Dabelic R Ahmed CM. IFN Signaling: How a Non-Canonical Model Led to the Development of IFN Mimetics. Front Immunol (2013) 4:202. doi: 10.3389/fimmu.2013.00202 23898330 PMC3722551

[13] Au-Yeung N Mandhana R Horvath CM. Transcriptional Regulation by STAT1 and STAT2 in the Interferon JAK-STAT Pathway. JAKSTAT (2013) 2(3):e23931. doi: 10.4161/jkst.23931 24069549 PMC3772101

[14] Stetson DB Mohrs M Reinhardt RL Baron JL Wang ZE Gapin L. Constitutive Cytokine mRNAs Mark Natural Killer (NK) and NK T Cells Poised for Rapid Effector Function. J Exp Med (2003) 198(7):1069–76. doi: 10.1084/jem.20030630 PMC2194220 14530376

[15] Lin Y Lu R Hou J Zhou GG Fu W. IFNgamma-Inducible CXCL10/CXCR3 Axis Alters the Sensitivity of HEp-2 Cells to Ionizing Radiation. Exp Cell Res (2021) 398(1):112–21. doi: 10.1016/j.yexcr.2020.112382 33253709

[16] Jorgovanovic D Song M Wang L Zhang Y. Roles of IFN-γ in Tumor Progression and Regression: A Review. Biomark Res (2020) 8:49. doi: 10.1186/s40364-020-00228-x 33005420 PMC7526126

[17] Pereiro P Forn-Cuni G Figueras A Novoa B. Pathogen-Dependent Role of Turbot (*Scophthalmus Maximus*) Interferon-Gamma. Fish Shellfish Immunol (2016) 59:25–35. doi: 10.1016/j.fsi.2016.10.021 27742586

[18] Yang Y Huang Y Yu Y Zhou S Wang S Yang M. Negative Regulation of the Innate Antiviral Immune Response by TRIM62 From Orange Spotted Grouper. Fish Shellfish Immunol (2016) 57:68–78. doi: 10.1016/j.fsi.2016.08.035 27539706

[19] Xiang Y Liu W Jia P Li Y Jin Y Chen L. Molecular Characterization and Expression Analysis of Interferon-Gamma in Black Seabream Acanthopagrus Schlegelii. Fish Shellfish Immunol (2017) 70:140–8. doi: 10.1016/j.fsi.2017.08.046 28870857

[20] Somamoto T Miura Y Nakanishi T Nakao M. Local and Systemic Adaptive Immune Responses Toward Viral Infection *via* Gills in Ginbuna Crucian Carp. Dev Comp Immunol (2015) 52(1):81–7. doi: 10.1016/j.dci.2015.04.016 25936589

[21] Zhen CW Yuan HW Tian GM Li Q Zhang SH Xu QQ. Immune Regulation of IFN-γ in Chinese Sturgeon. J Fish China (2020) 44(9):1539–48. doi: 10.11964/jfc.20200712346

[22] Bolger AM Lohse M Usadel B. Trimmomatic: A Flexible Trimmer for Illumina Sequence Data. Bioinformatics (2014) 30(15):2114–20. doi: 10.1093/bioinformatics/btu170 PMC4103590 24695404

[23] Grabherr MG Haas BJ Yassour M Levin JZ Thompson DA Amit I. Trinity: Reconstructing a Full-Length Transcriptome Without a Genome From RNA-Seq Data. Nat Biotechnol (2011) 29(7):644–52. doi: 10.1038/nbt.1883 PMC3571712 21572440

[24] Li W Jaroszewski L Godzik A. Clustering of Highly Homologous Sequences to Reduce the Size of Large Protein Databases. Bioinformatics (2001) 17(3):282–3. doi: 10.1093/bioinformatics/17.3.282 11294794

[25] Simão FA Waterhouse RM Ioannidis P Kriventseva EV Zdobnov EM. BUSCO: Assessing Genome Assembly and Annotation Completeness With Single-Copy Orthologs. Bioinformatics (2015) 31(19):3210–2. doi: 10.1093/bioinformatics/btv351 26059717

[26] Buchfink B Xie C Huson DH. Fast and Sensitive Protein Alignment Using Diamond. Nat Methods (2015) 12(1):59–60. doi: 10.1038/nmeth.3176 25402007

[27] Mistry J Finn RD Eddy SR Bateman A Punta M. Challenges in Homology Search: HMMER3 and Convergent Evolution of Coiled-Coil Regions. Nucleic Acids Res (2013) 41(12):e121–1. doi: 10.1093/nar/gkt263 PMC3695513 23598997

[28] Mistry J Chuguransky S Williams L Qureshi M Salazar GA Sonnhammer ELL. Pfam: The Protein Families Database in 2021. Nucleic Acids Res (2020) 49(D1):D412–9. doi: 10.1093/nar/gkaa913 PMC7779014 33125078

[29] Langmead B Salzberg SL. Fast Gapped-Read Alignment With Bowtie 2. Nat Methods (2012) 9(4):357–9. doi: 10.1038/nmeth.1923 PMC3322381 22388286

[30] Roberts A. Ambiguous Fragment Assignment for High-Throughput Sequencing Experiments. PhD Dissertation. Berkeley: University of California (2013). Available at: https://escholarship.org/uc/item/7zx1s4hr.

[31] R Core Team. R: A Language and Environment for Statistical Computing. Vienna, Austria: R Foundation for Statistical Computing (2020). Available at: https://www.R-project.org/.

[32] Qi ZT Wu P Zhang QH Wei YC Wang ZS Qiu M. Transcriptome Analysis of Soiny Mullet (*Liza Haematocheila*) Spleen in Response to Streptococcus Dysgalactiae. Fish Shellfish Immunol (2016) 49:194–204. doi: 10.1016/j.fsi.2015.12.029 26707943

[33] Li G Zhao Y Wang J Liu B Sun X Guo S. Transcriptome Profiling of Developing Spleen Tissue and Discovery of Immune-Related Genes in Grass Carp (*Ctenopharyngodon Idella*). Fish Shellfish Immunol (2017) 60:400–10. doi: 10.1016/j.fsi.2016.12.012 27965162

[34] Maekawa S Byadgi O Chen YC Aoki T Takeyama H Yoshida T. Transcriptome Analysis of Immune Response Against Vibrio Harveyi Infection in Orange-Spotted Grouper (*Epinephelus Coioides*). Fish Shellfish Immunol (2017) 70:628–37. doi: 10.1016/j.fsi.2017.09.052/ 28939531

[35] Amparyup P Charoensapsri W Samaluka N Chumtong P Yocawibun P Imjongjirak C. Transcriptome Analysis Identifies Immune-Related Genes and Antimicrobial Peptides in Siamese Fighting Fish (*Betta Splendens*). Fish Shellfish Immunol (2020) 99:403–13. doi: 10.1016/j.fsi.2020.02.030 32081810

[36] Rowland RRR Joan L Jack D. Control of Porcine Reproductive and Respiratory Syndrome (PRRS) Through Genetic Improvements in Disease Resistance and Tolerance. Front Genet (2012) 3:260. doi: 10.3389/fgene.2012.00260 23403935 PMC3565991

[37] Wang G. Effects of TLR2 and IFN-γ on Phagocyte Resistance to Listeria Infection. PhD Dissertation. Jinan: Shandong University (2018). Available at: https://cdmd.cnki.com.cn/Article/CDMD-10422-1019013315.htm.

[38] Saitoh T Satoh T Yamamoto N Uematsu S Takeuchi O Kawai T. Antiviral Protein Viperin Promotes Toll-Like Receptor 7 and Toll-Like Receptor 9-Mediated Type I Interferon Production in Plasmacytoid Dendritic Cells. Immunity (2011) 34(3):285–7. doi: 10.1016/j.immuni.2011.03.010 21435586

[39] Reikine S Nguyen JB Modis Y. Pattern Recognition and Signaling Mechanisms of RIG-I and MDA5. Front Immunol (2014) 5:2014.00342. doi: 10.3389/fimmu.2014.00342 PMC4107945 25101084

[40] Loo YM Gale M Jr. Immune Signaling by RIG-I-Like Receptors. Immunity (2011) 34(5):680–92. doi: 10.1016/j.immuni.2011.05.003 PMC3177755 21616437

[41] Müller M Carte S Hofer MJ and Campbell IL. The Chemokine Receptor CXCR3 and Its Ligands CXCL9, CXCL10 and CXCL11 in Neuroimmunity–A Tale of Conflict and Conundrum. Neuropathology Appl Neurobiol (2010) 36(5):368–87. doi: 10.1111/j.1365-2990.2010.01089.x 20487305

[42] Laing KJ Bols N Secombes CJ. A CXC Chemokine Sequence Isolated From the Rainbow Trout *Oncorhynchus Mykiss* Resembles the Closely Related Interferon-Gamma-Inducible Chemokines CXCL9, CXCL10 and CXCL11. Eur Cytokine Netw (2002) 13(4):462–73. 12517732

