# Supplementary material for: Transcriptome Sequencing Reveals the Antiviral Innate Immunity by IFN-γ in Chinese Sturgeon Macrophages
Source: Front Immunol. 2022 Mar 17;13:854689. doi: 10.3389/fimmu.2022.854689 (PMC8967981; doi:10.3389/fimmu.2022.854689)
Supplement: Supplementary Table 1 — Nucleotide sequences of primers. [file DataSheet_1.docx]

# Supplementary Tables

| Primers | Sequence 5′-3′ |
| --- | --- |
| IFNe1-qF/R | GCTGCTGCTCATCGCTAGGAT/TGTTCCATGCTGTCACCGAGTC |
| IFNe2-qF/R | AACCTTCACCGCACGACTTCT/TGACGCTTCAGACTCCTGTTCT |
| IFNe3-qF/R | GGTAGCCGTGAGCCAGAGTT/GGTAGCAGTTGCAGGACTCAGT |
| IFNγ-qF/R | GGCTGGTGGAGGTCCATTGTT/ACGCTGATCTTCAGTGGTGTCT |
| IRF1-qF/R | CCACAGCCGACAGCACAAAC/ TCAGGAAACCTTTGCCATTA |
| IRF2-qF/R | TCATCAGCAACCCCCCCGAC/ TTGCCTTTGTGTCGTCATCG |
| IRF3-qF/R | ACCCCTCCCCCTTGCTTGATA/ GGTTGGTGTTGTAAATCTCCG |
| IRF7-qF/R | ACTCCTCCTGCCTGATTTGA/ GTGTCCCGTAGATGCCCTTT |
| *AS*β-actin-qF/R | CCTTCTTGGGTATGGAATCTTGC/CAGAGTATTTACGCTCAGGTGGG |

**Supplementary Table 1.** Nucleotide sequences of primers.

| Sample | AM1 | AM2 | AM3 | AMC1 | AMC2 | AMC3 |
| --- | --- | --- | --- | --- | --- | --- |
| Raw_reads | 89138792 | 88627374 | 90354258 | 94110732 | 88326522 | 97939542 |
| Raw_bases | 13370818800 | 13294106100 | 13553138700 | 14116609800 | 13248938300 | 14690931300 |
| Clean_reads | 87053982 | 96675080 | 88386552 | 91909484 | 86255242 | 95731264 |
| Clean_bases | 12237053672 | 12239380102 | 12465165380 | 12873603160 | 12002996276 | 13401973749 |
| Valid_bases | 91.52% | 92.07% | 91.97% | 91.19% | 90.06% | 91.23% |
| Q30 | 93.01% | 93.26% | 93.21% | 92.99% | 93.11% | 93.28% |
| GC | 45.86% | 46.11% | 46.24% | 46.51% | 46.48% | 46.56% |

**Supplementary Table 2.** Transcriptome data statistics.

| Term | All（>= 300 bp） | > = 500 bp | >=1000 bp | N 50 | Total_Length | Max_Length | Min_Length | Average_Length |
| --- | --- | --- | --- | --- | --- | --- | --- | --- |
| Unigenne | 88879 | 52091 | 27054 | 1727 | 93916393 | 26838 | 301 | 1056.68 |

**Supplementary Table 3.** Clean reads assembly statistics.

| **Anno Database** | **Annotated Number** | **300≤length<1000** | **length≥1000** |
| --- | --- | --- | --- |
| NR | 27679（31.14 %） | 10521（11.84 %） | 17158（19.30 %） |
| Swiss-Prot | 23190（26.09 %） | 7638（8.59 %） | 15552（17.50 %） |
| KEGG | 16011（18.01 %） | 5392（6.07 %） | 10619（11.95 %） |
| KOG | 17081（19.22 %） | 5352（6.02 %） | 11729（13.20 %） |
| eggNOG | 25138（28.28 %） | 8870（9.98 %） | 16268（18.30 %） |
| GO | 20900（23.52 %） | 6810（7.66 %） | 14090（15.85 %） |
| Pfam | 16700（18.79 %） | 3747（4.22 %） | 12953（14.57 %） |

**Supplementary Table 4.** Statistical table of database annotation.

| Pathway | DEGs numbers（548） | Percent of DEGs |
| --- | --- | --- |
| Influenza A | 66 | 12.0438% |
| Cytokine-cytokine receptor interaction | 50 | 9.1241% |
| Herpes simplex infection | 62 | 11.3139% |
| RIG-I-like receptor signaling pathway | 35 | 6.3869% |
| Pertussis | 37 | 6.7518% |
| Cytosolic DNA-sensing pathway | 28 | 5.1095% |
| Hepatitis B | 50 | 9.1424% |
| TNF signaling pathway | 41 | 7.4818% |
| Measles | 42 | 7.6642% |
| Toll-like receptor signaling pathway | 32 | 5.8394% |
| NOD-like receptor signaling pathway | 27 | 4.927% |
| Cell adhesion molecules （CAMs） | 26 | 4.7445% |
| AGE-RAGE signaling pathway in diabetic complications | 32 | 5.8394% |
| Rheumatoid arthritis | 25 | 4.562% |
| Toxoplasmosis | 31 | 5.6569% |
| Legionellosis | 23 | 4.1971% |
| Jak-STAT signaling pathway | 30 | 5.4745% |
| Malaria | 16 | 2.9197 |
| Apoptosis-multiple species | 14 | 2.5547% |
| Hepatitis C | 41 | 7.4818% |

**Supplementary Table 5.** Pathway enrichment analysis of AM-vs-AMC, statistical table of KEGG enrichment's top 20 signal pathways.

| Category/Gene name | Description | Log_2_ Fold change | *P*-value |
| --- | --- | --- | --- |
| **TNF signaling pathway** | | | |
| MMP9 | matrix metalloproteinase-9 （gelatinase B） | -4.20314 | 0.004494 |
| MMP2 | matrix metalloproteinase-2 （gelatinase A） | -4.03909 | 0.228658 |
| TNFA | tumor necrosis factor superfamily, member 2 | 2.129854 | 1.41E-05 |
| TNFRSF6B | tumor necrosis factor receptor superfamily member 6B | 1.787346 | 2.16E-09 |
| MLKL | mixed lineage kinase domain-like | 1.688808 | 2.15E-10 |
| TRAF2 | TNF receptor-associated factor 2 | 1.50877 | 5.85E-05 |
| **Chemokine signaling pathway** | | | |
| CXCL10 | C-X-C motif chemokine 10 | 3.773626 | 3.16E-08 |
| CCR4 | C-C chemokine receptor type 4 | -1.82094 | 0.271967 |
| IL8RB, CXCR2 | C-X-C chemokine receptor type 2 | -1.62799 | 0.001572 |
| CXCL11 | C-X-C motif chemokine 11 | 2.914513 | 8.31E-05 |
| CCL20 | C-C motif chemokine 20 | 2.663286 | 0.120928 |
| CMKLR1 | chemokine-like receptor 1 | 2.298737 | 6.09E-05 |
| IFNAR2 | interferon receptor 2 | 2.017802 | 3.87E-05 |
| CCL28 | C-C motif chemokine 28 | 2.041165 | 5.91E-06 |
| IL15RA | Interleukin 15 receptor alpha | 1.912255 | 2.41E-10 |
| IL7 | interleukin 7 | 1.68329 | 2.47E-08 |
| IL2RG | interleukin 2 receptor gamma | 1.619531 | 0.000237 |
| IL10RB | interleukin 10 receptor beta | 1.513509 | 4.07E-05 |
| DAPP1, BAM32 | dual adapter for phosphotyrosine and 3-phosphotyrosine and 3-phosphoinositide | -2.70954 | 0.012518 |
| **RIG-I-like receptor signaling pathway** | | | |
| DDX58, | ATP-dependent RNA helicase DDX58 | 3.077863 | 0.000517 |
| IFIH1, MDA5 | interferon-induced helicase C domain-containing protein 1 | 2.502528 | 1.49E-07 |
| IRF7 | interferon regulatory factor 7 | 2.469555 | 4.52E-06 |
| IRF1 | interferon regulatory factor 1 | 2.274568 | 6.50E-05 |
| IFIH1, MDA5 | interferon-induced helicase C domain-containing protein 1 | 2.215835 | 5.55E-06 |
| IRF4 | interferon regulatory factor 4 | 1.876041 | 5.77E-05 |
| IRF3 | interferon regulatory factor 3 | 1.845416 | 0.000262 |
| IRF8 | interferon regulatory factor 8 | 1.501915 | 0.000242 |
| **Toll-like receptor signaling pathway** | | | |
| TLR13 | toll-like receptor 13 | 2.609135 | 0.134387 |
| IL6 | interleukin 6 | 2.349548 | 0.000147 |
| CD86 | CD86 antigen | 1.375283 | 6.90E-05 |
| **NOD-like receptor signaling pathway** | | | |
| CARD8, CARDINAL | caspase recruitment domain-containing protein 8 | 1.715319 | 7.69E-07 |
| SUGT1, SGT1 | suppressor of G2 allele of SKP1 | 1.659499 | 8.47E-06 |
| NFKBIE | NF-kappa-B inhibitor epsilon | 1.366159 | 2.43E-05 |
| **Others** | | | |
| STAT1 | signal transducer and activator of transcription 1 | 2.080241 | 0.000956 |
| JAK2, CSF3 | Janus kinase 2granulocyte colony-stimulating factor | 2.029884 | 5.77E-05 |
| CASP3 | caspase 3 | 1.7805 | 0.000108 |
| TRIM25, EFP | tripartite motif-containing protein 25 | 1.689302 | 2.24E-05 |

**Supplementary Table 6.** Immune signal pathway and screening of differential genes.
